# A randomised-controlled trial of two educational modes for undergraduate evidence-based medicine learning in Asia

**DOI:** 10.1186/1472-6920-9-63

**Published:** 2009-09-29

**Authors:** Janice M Johnston, C Mary Schooling, Gabriel M Leung

**Affiliations:** 1 School of Public Health, Li Ka Shing Faculty of Medicine, The University of Hong Kong, Faculty of Medicine Building, 21 Sassoon Road, Pokfulam, Hong Kong, PR China

## Abstract

**Background:**

As the overall evidence for the effectiveness of teaching of evidence based medicine (EBM) is not strong, and the impact of cultural and societal influences on teaching method is poorly understood, we undertook a randomised-controlled trial to test the effectiveness and learning satisfaction with two different EBM teaching methods (usual teaching vs. problem based learning (PBL)) for undergraduate medical students.

**Methods:**

A mixed methods study that included a randomised-controlled crossover trial with two intervention arms (usual teaching and PBL) and a nested qualitative study with focus groups to explore student perceptions of learning and to assess the effectiveness and utility of the two teaching methods.

All 129 second-year medical students at the University of Hong Kong in 2007.

The main outcomes measures were attitudes towards EBM; personal application and current use of EBM; EBM knowledge; future use of EBM.

**Results:**

PBL was less effective at imparting knowledge than usual teaching consisting of a lecture followed by a group tutorial. After usual teaching students showed improvement in scores for 'attitudes towards EBM', 'personal application and current use of EBM' and 'EBM knowledge, which were not evident after PBL. In contrast to the usual teaching, students found PBL difficult as they lacked the statistical knowledge necessary to support discussion, failed to understand core concepts, and lost direction.

**Conclusion:**

The evidence presented here would suggest that the teaching of EBM within an Asian environment should adopt a format that facilitates both the acquisition of knowledge and encourages enquiry.

## Background

Evidence-based medicine (EBM) has been defined as the "conscientious, explicit and judicious use of current best evidence in making decisions about the care of individual patients"[1]. While the teaching of EBM has most frequently been located in residency, internship, postgraduate and continuing education programmes, information management and decision making skills, core constituents of evidence-based medicine, are also key competencies for medical students in their formative years [2]. There remains, however, a lack of consensus as to the best teaching and learning methods for integrating EBM (or its precursors of statistics and epidemiology) into an undergraduate medical curriculum [2-4]. Though there is some evidence from western populations that students in a problem based learning (PBL) curriculum become better at problem solving and self-directed learning than those in a traditional curriculum; [5-8] others present contrasting evidence of the effectiveness of PBL [9-11]. Overall, there is little evidence to support the utility of PBL for EBM learning or the generalisability and applicability of these findings across cultures.

The PBL constructivist model holds that students do not passively absorb new information but build new ideas and concepts on pre-existing knowledge [12]. However, this open communication and Socratic learning style, where students are expected to question their own and others' beliefs as well as to evaluate and esteem self-generated knowledge, adopted by PBL and EBM may pose problems for some Asian students [13]. In many Asian societies learning is closely tied to hard work, undertaken for a utilitarian purpose where information and knowledge are to be acquired, rather than evaluated [14]. The emphasis on harmony and group coherence in many Asian societies as well as the social, cultural, and educational environments most likely influence beliefs regarding the acquisition of knowledge and the acceptability of self-regulated learning [15].

In contrast to other students, medical students express a more dualistic view of knowledge acquisition, i.e. perceived largely as right or wrong or as true or false [16]. Such beliefs, that knowledge functions in this strictly dualistic way, affect both the motivational and strategic components of self-regulated learning. Aspects of these beliefs are thought to vary between societies with more or less collectivistic cultures [17] influencing culturally regulated approaches to study and learning [5]. As such medical students, particularly those in Asia, may find incongruence between their epistemological beliefs, where knowledge is perceived as acquired from others, and instructional style such as PBL, where knowledge is acquired through exploration and enquiry, an impediment to learning.

Educational research to support teaching and learning is important, both for its practical relevance but also to bring teaching and learning in line with current pedagogy including lifelong self-regulated learning and social learning theory [17]. As the overall evidence of teaching effectiveness for EBM is not strong, and the impact of cultural and societal influences on teaching methods are poorly understood, we undertook a randomised-controlled trial with undergraduate medical students to test the effectiveness and learning satisfaction of two different teaching methods (usual teaching vs. PBL) for EBM.

## Methods

### Study participants

All second-year medical students (N = 129) attending the University of Hong Kong's five-year undergraduate medical program were included in the study. The trial was explained in detail to the year group. Students were assured of the anonymity and confidentiality of personal information for all responses throughout the study period.

We undertook a mixed methods study that included a randomised-controlled crossover trial with two intervention arms, usual teaching and problem based learning, and a nested qualitative study with focus groups to explore student perceptions of learning. To ensure that all students had the same overall learning experience, we used a cross-over design, where students either had usual teaching followed by PBL or PBL followed by usual teaching.

A two-stage randomisation strategy was adopted. First, students were divided into 13 standard learning groups of approximately equal size (9-10 students each) by the Faculty of Medicine's Medical Education Unit, independent of the research team. Assignment of students and the randomisation process were concealed from both the participants and investigators. Seven such learning groups were randomly assigned to the usual teaching intervention arm in the first half of the trial followed by the PBL intervention in the second half and six groups were randomly assigned to the PBL intervention arm followed by usual teaching intervention.

Focus groups, using purposive sampling [18,19] allowed us to explore the different learning experiences of the students in the two intervention arms. Students were selected to maximize sample variation on criteria judged as likely to influence preferences for different teaching methods, as well as attitudes to EBM learning. These criteria were gender, age, locale of secondary schooling, or prior degree. Of the 129 eligible students, 25 were individually approached and invited to participate, 15 agreed and attended the baseline focus group, 9 attended the first focus group session and 5 the second. Informed consent was obtained verbally. The tight system block schedule led to little flexibility in scheduling focus group sessions and contributed to the student non-response to these sessions. In addition students who refused or did not attend the focus group sessions stated they were either too busy or lacked interest. Provision of a coffee shop voucher encouraged participation in each focus group session.

**Figure 1:**
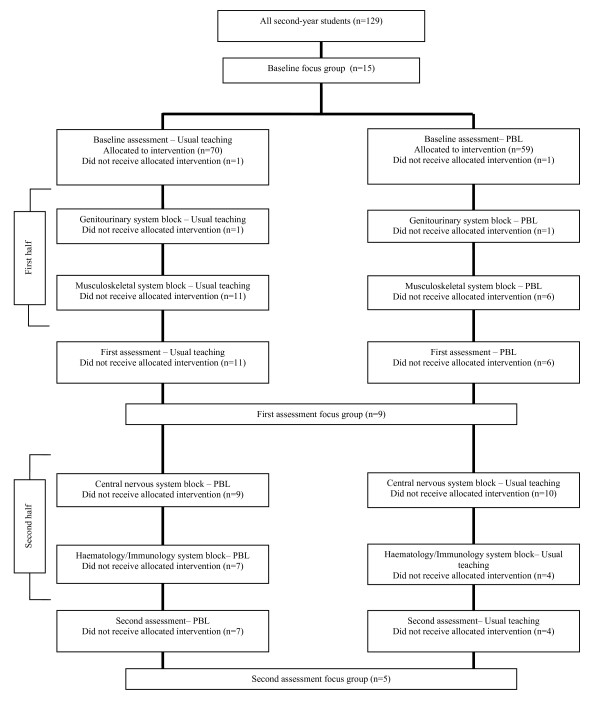
**Study organisation**. Note: Knowledge, attitude and behaviour questionnaire

Figure 1 shows the progression of students through the different stages of the trial.

### Educational interventions

The EBM curriculum spans five out of six body system blocks in the second year; four body system blocks were included in this trial. The teaching programme is self-contained within each body system block, and includes 2 two-hour sessions per body system block, so students had two sets of 2 two-hour sessions of one intervention arm in the first half of the trial and another two sets of 2 two-hour sessions of the other intervention in the second half of the trial. The EBM programme takes an integrated approach to applying the 5A's of EBM, i.e. assess the patient, ask an answerable clinical question, acquire the evidence, appraise the evidence, and apply the evidence back to the clinical situation, within each body system block.

The usual teaching arm began with a 2-hour session for all assigned students. The students were first shown a video consultation where a doctor interviewed a real patient and took a clinical history. A faculty member then reviewed the salient points from the patient encounter on the video, identified learning points, collectively formulated a specific answerable clinical question, demonstrated a targeted literature search in real time online and found a suitable original article that would be critically appraised in the subsequent group tutorial (~20 students). Finally, the session concluded with a statistical primer on key concepts that would be useful in the critical appraisal exercise.

In the PBL arm, using a paper case format, students in small groups (9-10 students each) met with a faculty tutor for the first 2-hour session and undertook essentially the same tasks as in the usual teaching arm. They watched the same video, identified learning points, formulated an answerable clinical question and carried out an online literature search for an original article.

For both arms, the student's task between the first and second sessions was to read, understand and critically appraise the chosen paper as well as to complete a statistical worksheet.

The content of the second 2-hour session was identical in both intervention arms. Students, following a set of guidance notes and facilitated by a faculty tutor, discussed and debated the relative merits and shortcomings of the paper, then they applied the discussion to the patient problem introduced in the video.

Table 1 summarises key features of the two teaching methods.

**Table 1:** Intervention comparison for usual and PBL teaching

	**EBP cycle**	**Usual**	**PBL**
**Session 1 **(2 hours)	Assess Ask	Whole class session Using an interactive teaching format led by a clinically qualified faculty member, students:-	Small group Using a PBL case format and facilitated by a faculty tutor (clinical or non-clinical), students:-

		1) Watch the video patient interview	
		2) Explore problems presented in the video, including the pathophysiology, presentation, history and physical findings	
		3) Set a PICO^1 ^question	
		4) Conduct on-line real time search for evidence to address the PICO question	
		5) Select the scientific paper for review in Session 2	
		
		Short lecture by statistical tutor	
		1) Brief lecture focused on statistics found in the paper	No statistical briefing

**Between Sessions**	Acquire	Between Sessions 1 and 2 students	
		1) Download the selected journal article from the course website.	
		2) Read the paper.	
		3) Complete the homework assignment.	

**Session 2 **(2 hours)	Appraise Apply	Using a set of guidelines and meeting in groups of 20 -- 30 divided into 2-3 groups meeting in one room and facilitated by a faculty tutor who circulates between students:-	Continuing with the PBL paper case with one faculty tutor (clinical or non clinical) per group (9-10) students:-
		
		1) Undertake a critical appraisal of the selected scientific paper	
		2) Draw conclusions about the validity and reliability of the data	
		3) Apply their conclusions to the patient as presented in the video in session 1	

Note: PICO^1^ = patient, intervention, comparison, outcome

### Measurement of outcomes

We defined, *a priori*, outcome indicators most relevant to the learning needs and objectives of second-year medical students, as assessed by self-report through a locally validated, standardised knowledge, attitude and behaviour (KAB) questionnaire [20].

The students completed the KAB questionnaire at baseline, at the first assessment, which was after the first two body system blocks (Genitourinary and Musculoskeletal) and at the second assessment, which was after two more body system blocks (Central Nervous and Haematology/Immunology) (Figure 1). KAB response rates were 97% at baseline, 88% at first assessment and 89% at second assessment.

Three focus groups were conducted, (one each at baseline, after the first and after the second assessment) (Figure 1) to explore student opinions about their previous experience with EBM teaching, as well as perceptions regarding usual teaching and PBL for EBM learning. Each focus group, facilitated by a teaching assistant, was conducted for 45 - 60 minutes in English, audio-taped and transcribed. Additional file 1 summarises the focus group guiding questions. Transcripts were carefully examined and all references to EBM learning were independently coded by JJ and the focus group facilitator by hand, any identified differences were resolved by consensus agreement. We used a constant comparative method of data analysis in order to explore for emergent themes. During the course of the study, categories and concepts arising from different transcripts were compared and contrasted to ensure they were mutually exclusive and to see how they clustered or connected. No new themes were identified in the final focus group.

### Data analysis

We used a generalised linear model to compare questionnaire item scores by teaching method at each assessment adjusted for the baseline score in the first half of the trial and for the first assessment score in the second half of the trial, from which we report mean differences in the change in score with 95% confidence intervals. We present these differences with PBL teaching as the reference, so that a positive difference indicates a greater improvement for usual teaching. All analyses were performed using SPSS v. 16 (SPSS Inc., Chicago, IL).

For the focus groups, all references to learning were coded by two independent coders to maximize the validity of the analysis, and the transcripts were explored to identify emerging themes, categories, and concepts until no new information was seen in the analysis.

Ethical approval was received from the HKU/HA West Cluster Institutional Review Board.

## Results

The distribution of measured baseline variables was balanced between the two interventions arms. (Table 2)

**Table 2:** Characteristics of students at baseline

**Characteristic**	**Usual teaching** **(n = 70)**	**PBL** **(n = 59)**
Age (years) *Mean (SD)*	20.04(1.45)	19.63(0.83)
Women *n (%)*	32(45.7)	24(40.7)
Factor scores *Mean (SD)*		
EBP knowledge^1^	4.47 (0.51)	4.56 (0.56)
Personal application and current use of EBP^2^	2.58 (0.58)	2.79 (0.66)
Future use of EBP^3^	3.86 (0.49)	3.87 (0.57)
Attitudes towards EBP^1^	3.14 (0.58)	3.03 (0.60)

Note: ^1^1 = Strongly disagree; 6 = Strongly agree

^2^1 = Never, 5 = Every day

^3^1 = Not at all, 6 = Completely

Table 3 shows significant differences in the adjusted mean changes in score by teaching method (usual teaching vs. PBL) for the primary learning outcomes after the first and second halves of the trial. Students 'crossed' over to the alternative intervention after the first half of the trial. In the first half, usual teaching was associated with significant improvement in scores for 'attitudes towards EBM' compared with PBL. In the second half, usual teaching was associated with significant improvement in scores for 'EBM knowledge' and 'personal application and current use of EBM' compared with PBL; although the improvement in 'attitudes towards EBM' was not significant, the response remained strong.

**Table 3:** Adjusted† mean differences in changes in score between assessments by teaching method with 95% confidence intervals.

		**Change in score between assessments**		
		
		**PBL group**	**Usual care group**	
	**Factors**		**Δ**	**95% CI‡**
Baseline to first assessment	EBP knowledge	reference	0.07	-0.30 to 0.45
(n = 109)	Personal application and current use of EBP	reference	0.01	-0.39 to 0.40
	Future use of EBP	reference	0.07	-0.31 to 0.45
	Attitudes towards EBP	reference	0.51 **	0.19 to 0.83**
				
First assessment to second assessment	EBP knowledge	reference	0.63 **	0.19 to 1.07**
(n = 102)	Personal application and current use of EBP	reference	0.43*	0.10 to 0.76*
	Future use of EBP	reference	0.07	-0.31 to 0.46
	Attitudes towards EBP	reference	0.27	-0.01 to 0.64

Note: † Mean difference adjusted for baseline factor score

‡ CI = confidence interval

*= p < 0.05; **= p < 0.001

Three main themes, 'learning skills and concepts', 'group process as an aid to learning' and 'role of the tutor' reflecting students attitudes towards and perceptions of learning in groups were identified in the focus groups. "Learning skills and concepts" reflects on the organisation and structure of the learning environment. In contrast to the usual teaching, students found the EBM PBL sessions difficult as they lacked the statistical knowledge necessary to support discussion, failed to understand the core concepts, and therefore lost direction. The limitations of minimally guided small groups [11] were reflected in the students comments illustrated as follows:-

"We need to learn something about the facts first - if we are not all prepared then efficiency in the small group is a disaster."

"Without a whole class lecture we do not have enough knowledge for the small group discussion."

In the theme 'group process as an aid to learning' in contrast to the 'usual teaching', the students as with others [9,10] described perceptions both in support of and contrast to PBL pedagogy. Although the PBL environment enhanced communications between members of the group, students used what was taught in lectures to direct their problem solving which is antithetical to the PBL hypothetico-deductive process. These are illustrated as follows:-

"Communications are easier in a small group. It is helpful to learn in a small group."

"For PBL, we will talk about what was being taught during lectures, finding something in addition to what the teachers have told us."

As reflected in the theme 'role of the tutor' students in EBM PBL defaulted to the tutor for in depth explanation and support more than in 'usual teaching', and were also more tutor dependent [11] than in usual teaching as illustrated as follows:-.

"EBP PBL depends more on the tutor than normal PBL."

"Tutors need to be more involved/interactive. It requires higher level of tutor skills."

"In EBM PBL it is easy to loose direction. Many concepts are not understood."

As medical school progress and assessment is very important to the students, they are highly motivated by their perceptions of efficient and effective learning. Overall students were frustrated by the EBM PBL learning process.

## Discussion

We found that PBL (self-directed learning) was less effective at imparting knowledge than the usual teaching programme (directed learning) of a lecture followed by a group tutorial. In contrast to that found by others [10] the in depth qualitative interviews revealed that the students were less satisfied with the PBL teaching method, perhaps because they found this constructivist educational model frustrating and inefficient, viewing it as the uninformed leading the ignorant. Consistent with the more collectivist approach where knowledge is acquired before leaning can begin, students identified a lack of lectures or background support as a core weakness of PBL. Students within the PBL programme, in the absence of prior teaching, could not derive facts from the discussion and thus became increasingly frustrated, findings which are consistent with others and reflect a failure of the hypothetico-deductive approach in developing reasoning skills [11]. The students were either unable or unwilling to take the time to decipher the statistical and epidemiological concepts in the paper without the direct intervention of the tutor, perpetuating an unwillingness to engage with the teaching materials. Our findings indicate that when facts are necessary for understanding these need to be provided either through a preceding lecture or within the associated text/or materials. The focus group discussions further clarified the students overall perception of each learning method, each with its pros and cons. Anecdotal evidence from tutor debriefings also indicated that in the absence of a prefacing statistical lecture students had difficulty with the appraisal and interpretation of the study findings and then applying the evidence to the clinical scenario.

In addition, clinical application and decision-making uncertainty further frustrated the students who sought defined answers to clinical questions. Such attitudes perhaps reflect a social and cultural learning environment that focuses on acquiring and transferring knowledge rather than generating hypotheses, new ideas or contradicting authority[13,14]. This approach to learning is antithetical to the aims of EBM and PBL. In contrast, others have demonstrated the ineffectiveness of lecture-based instruction to equip students with the integrated knowledge that is necessary for effective problem solving. However, our findings that a collectivist approach remains a cultural attribute in a post-modern Asian setting, together with the student's dualistic view of knowledge acquisition, supports a more culturally relevant approach to EBM learning.

The evidence presented here would suggest that the teaching of EBM within an Asian environment should adopt a format that facilitates both the acquisition of knowledge and encourages enquiry, comprising a prefacing lecture followed by a small group PBL session. In an Asian setting and perhaps elsewhere, small groups may need to reject the minimal guidance directive advocated for PBL curricula substituting instead small group tutors who have the necessary process and content expertise to identify misconceptions, identify problems, and provide immediate, content specific feedback to correct misunderstand or misconceptions [11]. In addition, we plan to include more interactive web based learning [18] to further EBM knowledge acquisition, deeper learning and to improve students skills in the application of research to the clinical setting. To provide a more substantive curriculum evaluation based on hard learning outcomes our future work will focus on more direct and long term measures of the student performance, and developing and measuring the problem solving and decision making skills which are necessary in a competent EBM practitioner.

## Limitations

Our study is one of a few randomised controlled trials of EBM learning in undergraduate students. We achieved an acceptable overall response rate for the questionnaire survey. Although non-response bias in the focus groups may have been a concern, purposive sampling was used to identify and recruit members to the focus groups and in the smallest groups representativeness was maintained. Although we were able to achieve allocation concealment, used validated and blinded outcome measures, standardized educational intervention, and had sufficient power to demonstrate a difference between the two study arms we were unable to blind the study participants and tutors, control for contamination between the study arms, or use the same tutor for all groups. While the crossover design may be seen as a weakness (as the effect of the intervention may not washout when crossing over) the study outcomes required comparison and were strengthened by this study design. This study only begins to investigate the challenge of assessing the impact of culture on evidence-based medicine in undergraduate medical students.

## Conclusion

Our randomised-controlled trial in an Asian setting indicated that undergraduate EBM teaching was most effectively implemented in a format that facilitates both the acquisition of knowledge and encourages enquiry. As such, our study also draws attention to the importance of locating teaching methods within their social and cultural context, so to take advantage of students existing epistemological beliefs.

## Competing interests

The authors declare that they have no competing interests.

## Authors' contributions

JJ carried out the study, contributed to the statistical analysis, shared in the writing of the manuscript, CMS performed the statistical analysis and shared in the writing of the manuscript, GML was responsible for the conceptual framework of the undergraduate EBM programme, advised on the analytic framework and shared in the writing of the manuscript. All authors read and approved the final manuscript.

## Pre-publication history

The pre-publication history for this paper can be accessed here:



## Supplementary Material

Additional file 1 Supplemental table S1 Click here for file
