# Supplementary material for: A randomised-controlled trial of two educational modes for undergraduate evidence-based medicine learning in Asia
Source: BMC Med Educ. 2009 Sep 29;9:63. doi: 10.1186/1472-6920-9-63 (PMC2761870; doi:10.1186/1472-6920-9-63)
Supplement: Additional file 1 — Supplemental table S1 [file 1472-6920-9-63-S1.DOC]

**Semi-structured interview guide**

| 1. Tell me about the challenges or problems that you encountered in your EBP sessions. |
| --- |
| 1. What did you find most useful about EBP learning? |
| 1. What did you find least useful about EBP learning? |
| 1. What aspects of EBP learning do you think will be most useful in facilitating your future care for your patients? |
| 1. Do you think you will use EBM in your future daily clinical practice as a result of this EBP learning? |
| 1. Which aspects in this learning opportunity enhanced or discouraged your intention to integrate EBM in your clinical practice? |
| 1. Do you have any other opinions about this EBP learning experience? |
